# Correlation of osteoarthritis or rheumatoid arthritis with bone mineral density in adults aged 20–59 years

**DOI:** 10.1186/s13018-021-02338-0

**Published:** 2021-03-15

**Authors:** Zhongxin Zhu, Gangfeng Hu, Fang Jin, Xiaocong Yao

**Affiliations:** 1 Department of Osteoporosis Care and Control, Xiaoshan Affiliated Hospital of Wenzhou Medical University, Hangzhou, 311200 Zhejiang China; 2 Clinical Research Center, Xiaoshan Affiliated Hospital of Wenzhou Medical University, Hangzhou, 311200 Zhejiang China

**Keywords:** Osteoarthritis, Degenerative arthritis, Rheumatoid arthritis, Bone health, NHANES

## Abstract

**Background:**

It is reported that osteoporosis commonly occurs among patients with rheumatoid arthritis (RA), whereas the association between osteoporosis and osteoarthritis (OA) remains controversial. Our aim in this study was to investigate the association between BMD, as a marker of osteoporosis, and OA and RA among adults 20−59 years of age, using a population-based sample from the National Health and Nutrition Examination Survey (NHANES).

**Methods:**

Our analysis was based on the NHANES data collected between 2011 and 2018. Data regarding arthritis status and the type of arthritis (OA or RA) were obtained from questionnaires. Lumbar BMD was measured by dual-energy X-ray absorptiometry. The association between OA, RA, and lumbar BMD was evaluated using logistic regression models. Subgroup analyses, stratified by gender and race, were performed. The association between duration of arthritis and lumbar BMD was also investigated.

**Results:**

A total of 11,094 adults were included in our study. Compared to the non-arthritis group, participants with OA had a higher lumbar BMD (*β* = 0.023, 95% CI 0.011–0.035), with no significant association between lumbar BMD and RA (*β* = 0.014, 95% CI − 0.003 to 0.031). On subgroup analyses stratified by gender, males with OA had a higher lumbar BMD compared to those without OA (*β* = 0.047, 95% CI 0.028–0.066). In females, OA was not associated with lumbar BMD (*β* = 0.007, 95% CI − 0.008 to 0.021). There was no association between lumbar BMD and RA in both males (*β* = 0.023, 95% CI − 0.003 to 0.048) and females (*β* = 0.008, 95% CI − 0.015 to 0.031). Duration of arthritis was not associated with lumbar BMD for both OA (*β* = − 0.0001, 95% CI − 0.0017 to 0.0015) and RA (*β* = 0.0006, 95% CI − 0.0012 to 0.0025).

**Conclusions:**

Lumbar BMD was associated with OA but not with RA. While a higher lumbar BMD was associated with OA in males, but not in females. Our findings may improve our understanding between OA, RA, and bone health.

## Introduction

Osteoporosis (OP), osteoarthritis (OA), and rheumatoid arthritis (RA) are pathologies of the musculoskeletal system that cause pain, movement impairments, and possibly permanent disability [1]. With aging of the general population, it is estimated that 1 in 4 adults in developed countries are affected by OP, OA, and/or RA [2, 3]. The pathophysiology of these three conditions is different; however, RA is an autoimmune disease of unknown etiology, associated with a homeostatic imbalance [4]. By contrast, OA is largely considered a biomechanical disorder of articular joints, with its onset and development being closely related to changes in inflammatory and catabolic functions of the body [5]. OP is a systemic disease associated with a marked loss of bone mineral density (BMD). As two notable silent rheumatic diseases, OA and OP have been included on the World Health Organization’s list of disabling disease [1, 6].

However, a clear association between OP and OA has not been clearly defined and remains an issue of controversy, while OP is commonly associated with RA [7–10]. Our aim in this study was to investigate the association between BMD, as a marker of OP, and OA and RA among adults 20–59 years of age, using a population-based sample from the National Health and Nutrition Examination Survey (NHANES).

## Methods

### Study population

The NHANES program is a series of surveys focusing on the health topics of the general population, of all ages, in the United States (US). A multistage, complex clustered, probability design is used for data collection and analysis, rather than being based on a simple random sample of the US population [11].

For our study, we used NHANES data collected between 2011 and 2018. The study population was restricted to adults 20–59 years of age (*n* = 14,934). From this eligible group, 3840 individuals were excluded for the following reasons: absence of an arthritis diagnosis (*n* = 23) or lumbar BMD measure (*n* = 3398), or a cancer diagnosis (*n* = 419) were excluded. A total of 11,094 individuals were included in our analysis.

Our study protocol was approved by the ethics review board of the National Center for Health Statistics (NCHS). Within the NHANES program, all participants have provided consent for the use of their anonymized information for research purposes [12].

### Arthritis

A diagnosis of arthritis was based on the medical conditions questionnaires collected by interview as part of the NHANES program. Participants were asked if they had ever been told by their doctor or other health professional that they had arthritis. If “yes”, they were asked to classify their arthritis diagnosis as OA, RA, psoriatic arthritis, other, or do not know/refuse to answer. The duration of arthritis was determined in years as the “age at screening” minus the “age at the time of diagnosis.”

### Lumbar BMD

The lumbar spine is one site that is typically evaluated for the assessment and treatment of OP, with BMD measurement at this site having been used as a marker of OP in clinical trials for over three decades [13]. BMD measures within the NHANES were obtained using dual-energy X-ray absorptiometry (DXA) scans, which is the most widely accepted method. Within the NHANES program, DXA scans were performed using a Hologic Discovery model A densitometers (Hologic, Inc., Bedford, Massachusetts), with data analyzed using Hologic APEX (version 4.0) software. Further details of the DXA examination protocol are available on the NHANES website.

### Collected data

The following demographic data were collected by interview questionnaires: age, gender, race, educational level, ratio of family income to poverty, vigorous recreational activities, and smoked history. Race was quantified as follows: non-Hispanic White, non-Hispanic Black, Mexican American, other Hispanic, and other race, including being multi-racial. Vigorous recreational activities was based on an individual’s self-reported answer to the following question: “Do you do any vigorous-intensity sports, fitness, or recreational activities that cause large increases in breathing or heart rate like running or basketball for at least 10 minutes continuously?.” A positive smoking history was defined as ≥ 100 cigarettes smoked in one’s life.

### Laboratory data

Biospecimens were collected for laboratory analysis to provide detailed information on each individual’s nutritional status and general health. Biospecimens were collected, processed, and stored in the mobile examination center until shipping to a laboratory for analysis. The following biomarkers were collected: blood urea nitrogen, total protein, total cholesterol, alkaline phosphatase, serum potassium, serum sodium, serum phosphorus, serum uric acid, and serum calcium.

### Statistical analysis

To assure national representation, we used weighted analyses as recommended by the analytical guidelines of the NCHS. The *P* value of the difference between individuals with and without arthritis was calculated using a weighted chi-squared test for categorical variables and a weighted linear regression model for continuous variables.

The association between arthritis (OA and RA) and lumbar BMD was examined using a multivariable logistic regression. Three models were constructed, as follows: model 1, no adjustment for covariates; model 2, adjusted for age, gender, and race; model 3, adjusted for age, gender, race, educational level, body mass index (BMI), ratio of family income to poverty, vigorous recreational activities, smoked history, blood urea nitrogen, total protein, total cholesterol, alkaline phosphatase, serum potassium, serum sodium, serum phosphorus, serum uric acid, and serum calcium were adjusted. In addition, in alignment with The Strengthening the Reporting of Observational Studies in Epidemiology (STROBE) guideline [14], we performed subgroup analyses, stratified by age and gender, to make better use of the data. We also performed multivariable logistic regression to explore the association between duration of OA and RA and lumbar BMD. All analyses were performed using package R (version 3.4.3, http://www.R-project.org) and EmpowerStats software (http://www.empowerstats.com). The significance level was set at 0.05 for all analyses.

## Results

### Study sample

The characteristics of the samples are presented in Table 1, with relevant features between the non-arthritis and arthritis group summarized, as follows. Compared to the non-arthritis group, the arthritis group was older (mean age 37.59 ± 11.34 years versus 47.99 ± 9.36 years), and had a higher proportion of women than men (46.17% versus 56.08%). Race, educational level, BMI, vigorous recreational activities, smoked history, blood urea nitrogen, total protein, total cholesterol, alkaline phosphatase, serum uric acid, serum sodium, and serum calcium were also significantly different between the two groups (*P* < 0.05).

**Table 1:** Characteristic of study sample with and without arthritis

	Arthritis (*n* = 1510)	Non-arthritis (*n* = 9584)	*P* value
Age (years)	47.99 ± 9.36	37.59 ± 11.34	< 0.001
Age groups			
20–29 years	5.97	30.31	< 0.001
30–39 years	12.38	26.12	
40–49 years	27.09	24.53	
50–59 years	54.56	19.04	
Gender (%)			
Male	43.92	53.83	< 0.001
Female	56.08	46.17	
Race (%)			
Mexican American	6.07	11.06	< 0.001
Other Hispanic	5.07	7.71	
Non-Hispanic White	68.41	58.80	
Non-Hispanic Black	12.47	12.32	
Other race—including multi-racial	7.97	10.11	
Educational level (%)			
Less than 9th grade	3.94	3.96	< 0.001
9th–11th grade	11.83	9.02	
High school graduate/GED or equivalent	22.99	21.67	
Some college or AA degree	35.13	32.47	
College graduate or above	26.11	32.87	
Not recorded	0	0.01	
Body mass index (kg/m2)	31.71 ± 8.00	28.56 ± 6.56	< 0.001
Ratio of family income to poverty	2.95 ± 1.72	2.94 ± 1.66	0.821
Vigorous recreational activities (%)			
Yes	19.99	36.63	< 0.001
No	80.01	63.37	
Smoked at least 100 cigarettes in life (%)			
Yes	51.38	38.63	< 0.001
No	48.59	61.35	
Not recorded	0.03	0.02	
Blood urea nitrogen (mmol/L)	4.82 ± 1.71	4.54 ± 1.49	< 0.001
Total protein (g/L)	70.60 ± 4.45	71.58 ± 4.29	< 0.001
Total cholesterol (mmol/L)	5.11 ± 1.04	4.91 ± 1.02	< 0.001
Alkaline phosphatase (U/L)	71.50 ± 22.57	66.32 ± 22.73	< 0.001
Serum uric acid(μmol/L)	323.79 ± 84.78	318.53 ± 80.72	0.019
Serum sodium (mmol/L)	139.12 ± 2.43	139.25 ± 2.21	0.037
Serum potassium (mmol/L)	3.95 ± 0.33	3.97 ± 0.31	0.122
Serum phosphorus (mmol/L)	1.20 ± 0.17	1.20 ± 0.18	0.397
Serum calcium (mmol/L)	2.34 ± 0.09	2.34 ± 0.08	0.013
Disease duration of arthritis (years)	9.20 ± 8.90	/	
Which type of arthritis was it? (%)			
Osteoarthritis or degenerative arthritis	41.61	/	/
Rheumatoid arthritis	17.45	/	
Psoriatic arthritis	2.73	/	
Other	0.17	/	
Do not know or refused	24.20	/	
Lumbar bone mineral density (g/cm^2^)	1.05 ± 0.16	1.04 ± 0.15	0.086

Mean ± SD for continuous variables: *P* value was calculated by weighted linear regression model

Percent for categorical variables: *P* value was calculated by weighted chi-square test

### Multiple regression model

As shown in Table 2, the association between arthritis and lumbar BMD was not significant in the unadjusted model (*β* = 0.007, 95% CI − 0.001 to 0.015). However, a significant association between arthritis and lumbar BMD was identified after adjusting for age, gender, and race (model 2: *β* = 0.017, 95% CI 0.008–0.025; model 3: *β* = 0.018, 95% CI 0.010–0.027). Compared to the non-arthritis group, individuals with OA or degenerative arthritis had a higher lumbar BMD (*β* = 0.023, 95% CI 0.011–0.035), with no significant association between the lumbar BMD and RA (*β* = 0.014, 95% CI − 0.003 to 0.031).

**Table 2:** Associations between arthritis and lumbar bone mineral density

	Model 1, *β* (95% CI, *P*)	Model 2, *β* (95% CI, *P*)	Model 3, *β* (95% CI, *P*)
Non-arthritis	Reference	Reference	Reference
Arthritis	0.007 (− 0.001, 0.015) 0.0861	0.017 (0.008, 0.025) 0.0001	0.018 (0.010, 0.027) < 0.0001
Non-arthritis	Reference	Reference	Reference
Osteoarthritis or degenerative arthritis	0.013 (0.001, 0.025) 0.0293	0.023 (0.012, 0.035) 0.0001	0.023 (0.011, 0.035) 0.0001
Rheumatoid arthritis	0.000 (− 0.017, 0.018) 0.9733	0.010 (− 0.007, 0.027) 0.2599	0.014 (− 0.003, 0.031) 0.1074
Psoriatic arthritis	0.005 (− 0.039, 0.049) 0.8177	0.020 (0.000, 0.039) 0.0445	0.024 (0.005, 0.043) 0.0133
Do not know or refused	0.000 (− 0.015, 0.015) 0.9712	0.008 (− 0.006, 0.023) 0.2665	0.011 (− 0.004, 0.026) 0.1350

*Model 1* no covariates were adjusted; *Model 2* age, gender, and race were adjusted; *Model 3* age, gender, race, educational level, body mass index, ratio of family income to poverty, vigorous recreational activities, smoked at least 100 cigarettes in life, blood urea nitrogen, total protein, total cholesterol, alkaline phosphatase, serum uric acid, serum sodium, serum potassium, serum phosphorus, and serum calcium were adjusted

### Subgroup analyses

In the subgroup analyses stratified by gender (Table 3), the lumbar BMD was higher among males with OA or degenerative arthritis than those without (*β* = 0.047, 95% CI 0.028–0.066). However, among females, lumbar BMD was not related to OA or degenerative arthritis (*β* = 0.007, 95% CI − 0.008 to 0.021). There was no association between lumbar BMD and RA in either males (*β* = 0.023, 95% CI − 0.003 to 0.048) or females (*β* = 0.008, 95% CI − 0.015 to 0.031).

**Table 3:** Subgroup analyses stratified by gender

	Model 1, *β* (95% CI, *P*)	Model 2, *β* (95% CI, *P*)	Model 3, *β* (95% CI, *P*)
Male			
Non-arthritis	Reference	Reference	Reference
Osteoarthritis or degenerative arthritis	0.045 (0.026, 0.065) < 0.0001	0.053 (0.034, 0.072) < 0.0001	0.047 (0.028, 0.066) < 0.0001
Rheumatoid arthritis	0.010 (− 0.016, 0.036) 0.4569	0.021 (− 0.005, 0.046) 0.1139	0.023 (− 0.003, 0.048) 0.0776
Female			
Non-arthritis	Reference	Reference	Reference
Osteoarthritis or degenerative arthritis	− 0.012 (− 0.026, 0.003) 0.1158	0.005 (− 0.010, 0.020) 0.4922	0.007 (− 0.008, 0.021) 0.3526
Rheumatoid arthritis	− 0.010 (− 0.034, 0.013) 0.3941	0.001 (− 0.022, 0.024) 0.9418	0.008 (− 0.015, 0.031) 0.5157

Adjusted for age, race, educational level, body mass index, ratio of family income to poverty, vigorous recreational activities, smoked at least 100 cigarettes in life, blood urea nitrogen, total protein, total cholesterol, alkaline phosphatase, serum uric acid, serum sodium, serum potassium, serum phosphorus, and serum calcium

In the subgroup analyses stratified by race (Table 4), non-Hispanic White adults with OA or degenerative arthritis had a higher lumbar BMD compared to those without (*β* = 0.014, 95% CI − 0.003 to 0.031).

**Table 4:** Subgroup analyses stratified by race

	Model 1, *β* (95% CI, *P*)	Model 2, *β* (95% CI, *P*)	Model 3, *β* (95% CI, *P*)
Non-Hispanic White			
Non-arthritis	Reference	Reference	Reference
Osteoarthritis or degenerative arthritis	0.009 (− 0.009, 0.026) 0.3439	0.018 (− 0.000, 0.036) 0.0501	0.019 (0.001, 0.037) 0.0382
Rheumatoid arthritis	− 0.015 (− 0.045, 0.015) 0.3222	− 0.007 (− 0.036, 0.023) 0.6699	0.001 (− 0.028, 0.031) 0.9232
Non-Hispanic Black			
Non-arthritis	Reference	Reference	Reference
Osteoarthritis or degenerative arthritis	− 0.020 (− 0.050, 0.009) 0.1806	0.003 (− 0.027, 0.034) 0.8340	0.007 (− 0.024, 0.037) 0.6611
Rheumatoid arthritis	0.008 (− 0.031, 0.046) 0.6947	0.032 (− 0.007, 0.071) 0.1105	0.032 (− 0.007, 0.071) 0.1035
Mexican American			
Non-arthritis	Reference	Reference	Reference
Osteoarthritis or degenerative arthritis	0.015 (− 0.025, 0.056) 0.4579	0.032 (− 0.008, 0.073) 0.1189	0.017 (− 0.023, 0.057) 0.4103
Rheumatoid arthritis	− 0.022 (− 0.061, 0.018) 0.2899	− 0.004 (− 0.044, 0.036) 0.8602	− 0.006 (− 0.046, 0.033) 0.7621

Adjusted for age, gender, educational level, body mass index, ratio of family income to poverty, vigorous recreational activities, smoked at least 100 cigarettes in life, blood urea nitrogen, total protein, total cholesterol, alkaline phosphatase, serum uric acid, serum sodium, serum potassium, serum phosphorus, and serum calcium

### Associations between disease duration and lumbar BMD

As shown in Table 5, there was no association between the lumbar BMD and either OA or degenerative arthritis (*β* = − 0.0001, 95% CI − 0.0017 to 0.0015) or RA (*β* = 0.0006, 95% CI − 0.0012 to 0.0025).

**Table 5:** Associations between disease duration of arthritis and lumbar bone mineral density

Disease duration of arthritis (years)	Model 1, *β* (95% CI) *P* value	Model 2, *β* (95% CI) *P* value	Model 3, *β* (95% CI) *P* value
Osteoarthritis or degenerative arthritis	0.0005 (− 0.0021, 0.0011) 0.5312	− 0.0003 (− 0.0020, 0.0013) 0.6746	− 0.0001 (− 0.0017, 0.0015) 0.8619
Rheumatoid arthritis	0.0013 (− 0.0005, 0.0032) 0.1596	0.0013 (− 0.0005, 0.0032) 0.1649	0.0006 (− 0.0012, 0.0025) 0.4979

Adjusted for age, race, educational level, body mass index, ratio of family income to poverty, vigorous recreational activities, smoked at least 100 cigarettes in life, blood urea nitrogen, total protein, total cholesterol, alkaline phosphatase, serum uric acid, serum sodium, serum potassium, serum phosphorus, and serum calcium

## Discussion

The main findings of our study were as follows: First is the positive association between a higher lumbar BMD and OA among males, but not among females. Second, there was no association between the lumbar BMD and RA, in both females and males.

The association between OP and OA has been an issue of controversy for a number of years. Both diseases depend on bone metabolism and are positively correlated with aging. In a study of 359 postmenopausal women 50–89 years of age, Povoroznyuk et al. [15] identified that women with symptomatic OA had a significantly higher lumbar BMD compared to controls. By contrast, a cross-sectional analysis of a Korean national survey reported a negative association between lumbar BMD and knee OA [16]. A recent prospective study provided strong evidence that high femoral neck BMD is a prognostic risk factor for the development of knee and hip radiographic OA [17]. In addition, higher BMD has been shown to reduce the risk of fractures among both men and women [18, 19]. The concomitant presence of OP and OA in patients with hip or spine OA has also been reported [20, 21]. In a study of 80 post-menopausal women with hand OA, including Heberden’s nodes which is a characteristic feature of primary generalized OA, and 80 age-matched women without OA, the authors found that primary generalized OA is not protective against osteoporosis [22]. The results of a cross-sectional study of 2855 individuals ≥ 40 years of age identified a significant association between elevated phalangeal BMD and radiographic knee OA among women but not men [23].

The biologic mechanism by which BMD influences OA has not been established. Previous statistically significant findings may result from uncontrolled and unmeasured confounding factors, such as skeletal growth factors [24], bone geometry [25, 26], bone morphology [27], and genetics [28]. A previous cohort analysis from the Framingham Study reported that a higher BMD decreased the risk of progression of radiographic knee OA, defined by the presence of osteophytes on radiographs [29]. The authors postulated that a higher BMD reduced the risk of joint space loss; however, once OA developed, a higher BMD might increase the risk of osteophyte formation. Other studies have also reported a positive association between BMD and osteophytes, with a negative association with joint space narrowing [30, 31]. In an animal experiment, knee OA induction by anterior cruciate ligament transection in young growing female rats induced greater bone loss in the weight-bearing bone than in non-weight-bearing bone during OA progression [32]. From a cellular point of view, OP patients exhibit an imbalance between the osteoblast and osteoclast activity [33], and dysregulation of bone remodeling contributes to the development of OA [34]. In our study, we identified a sex-specific difference in the association between lumbar BMD and OA. One possible explanation is that higher BMI and greater weight-bearing activities, which are more likely in men than in women, might both increase the risk of damage to articular cartilage leading to OA and also be beneficial to the preservation of bone mass.

The absence of an association between OP and RA may be a result of multiple factors. Of note, a link between OP and RA has previously been postulated, with this association being mediated via several mechanisms, including pro-inflammatory state, glucocorticoids use, low level of physical activity, and the classic risk factors for OP [35]. However, in a cross-sectional study of 152 Korean adults ≥ 50 years of age, Kweon et al. [36] found no significant difference in lumbar BMD between patients with and without RA. In a study of 138 postmenopausal women with RA, Mori et al. [37] found that disease duration was significantly related to BMD using multivariate linear regression analyses. In contrast, a study of 76 patients with RA identified a lower than expected BMD in patients in the first decade of their RA disease compared to reference population [38]. In a study of 299 Korean female patients with RA, Lee et al. [39] found no significant association between disease duration of RA and BMD. In our own analysis, we did not identify a significant association between BMD and RA, or disease duration of RA, and this both in males and females. Differences across studies are likely attributable to variations among studies, including demographic characteristics, sample size, study design, and confounding variables controlled for.

**Fig. 1:**
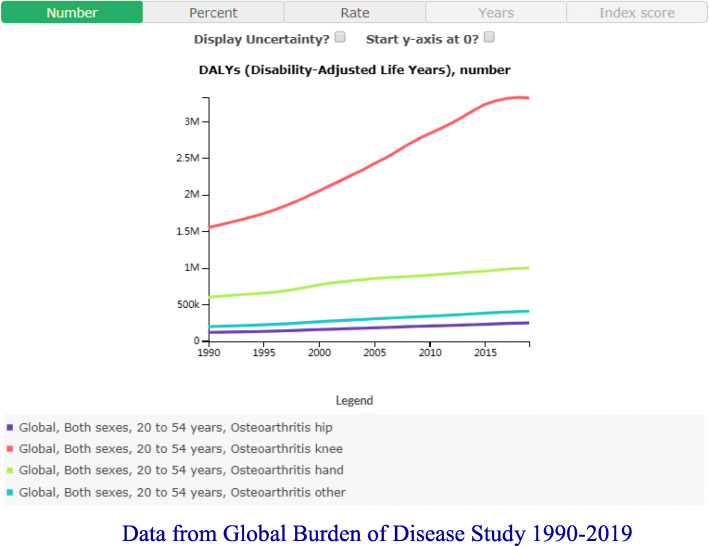
Data from Global Burden of Disease Study 1990–2019

The strengths of our study include a population-based sample with a wide age range that is generalizable to a community population, subgroup analyses for sensitivity test, and adjustment for many potential confounders. However, the limitations of our study also need to be acknowledged. First, due to the cross-sectional design of our study, we were unable to elucidate the causal relationship between arthritis and BMD. Longitudinal studies investigating the causality between them are needed. Second, the diagnosis of arthritis was based on patients’ self-report which may lead to bias. However, the consistency between self-reported arthritis and clinical confirmation has previously been documented [40, 41]. Third, the missing information on different sites of arthritis precludes us to estimate the associations between OA, RA, and BMD at specific sites. For example, primary osteoarthritis includes nonuniform joint space loss, osteophyte formation, cyst formation, and subchondral sclerosis at the lumbar spine which might increase the DXA measures of lumbar BMD, which is a considerable confounding factor in our study. However, the data from the Global Burden of Disease Study indicated that knee OA accounts for the vast majority of young and middle-aged adults [42] (Fig. 1). Fourth, our study results cannot be generalized as we excluded populations with special health concerns, such as individuals with a history of cancer. Finally, there might be other confounding factors we did not control for in our study, such as the use of glucocorticoids used for the treatment of RA. We do note, however, a previous study which did not identify a significant association between the cumulative glucocorticoid dose and BMD after adjustment for confounding variables [39]. The results of a population-based study similarly showed no significant difference in BMD between patients with RA treated with corticosteroids and a non-steroid group, indicative that an independent effect of corticosteroids on BMD is likely negligible [43].

In summary, lumbar BMD was associated with OA but not RA. Moreover, we identified a sex-specific effect, with a higher lumbar BMD associated with OA in males, but not in females. Our findings may improve our understanding between OA, RA, and bone health. Additional studies examining the association between BMD and OA and RA are warranted to confirm our findings.

## Abbreviations

OP: Osteoporosis
OA: Osteoarthritis
RA: Rheumatoid arthritis
BMD: Bone mineral density
NHANES: The National Health and Nutrition Examination Survey
US: The United States
NCHS: The National Center for Health Statistics
DXA: Dual-energy X-ray absorptiometry
BMI: Body mass index
STROBE: The Strengthening the Reporting of Observational Studies in Epidemiology
